# Integrative post-GWAS analysis prioritizes immune regulatory pathways and candidate effector signals in systemic lupus erythematosus

**DOI:** 10.1016/j.jtauto.2026.100399

**Published:** 2026-09-03

**Authors:** Vijayachitra Modhukur, Masuma Khatun, Naisarg Patel, Sajitha Lulu S, Prakash Lingasamy, Andres Salumets

**Affiliations:** a Celvia CC AS, Tartu, Estonia; b Department of Obstetrics and Gynecology, Institute of Clinical Medicine, University of Tartu, Tartu, Estonia; c Department of Obstetrics and Gynecology, Helsinki University Hospital and University of Helsinki, Helsinki, Finland; d Integrative Multiomics Lab, School of Bio Sciences and Technology, Vellore Institute of Technology, Vellore, India; e Laboratory of Precision and Nanomedicine, Institute of Biomedicine and Translational Medicine, University of Tartu, Tartu, Estonia; f Nalam Biosciences OÜ, Tartu, Estonia; g Division of Obstetrics and Gynaecology, Department of Clinical Science, Intervention and Technology (CLINTEC), Karolinska Institutet, Karolinska University Hospital, Stockholm, Sweden

**Keywords:** Systemic lupus erythematosus, GWAS, Post-GWAS, LAVA, Colocalization, *CLIC1*, Interferon signalling, JAK-STAT, Therapeutic prioritization

## Abstract

**Background:**

Systemic lupus erythematosus (SLE) has a complex polygenic architecture, but translating genome-wide association signals into biologically interpretable candidates remains challenging. We applied an integrative post-GWAS framework to refine SLE-associated loci and prioritize candidate regulatory mechanisms.

**Methods:**

European-ancestry SLE GWAS summary statistics from FinnGen and Bentham et al. were meta-analysed, comprising 8417 cases and 354,277 controls. After quality filtering, 6,782,131 SNPs were retained. Downstream analyses included LAVA regional prioritization, Bayesian colocalization with GTEx v8 whole-blood and spleen eQTLs, independent replication in the Julià et al. Spanish cohort, pathway enrichment, bivariate LAVA cross-trait local genetic correlation, and therapeutic annotation.

**Results:**

The discovery meta-analysis identified 46 genome-wide significant SLE-associated loci, including putative novel signals requiring database/literature qualification. LAVA identified 14 candidate index variants across 12 high-confidence regions, of which nine index variants were retained as the primary prioritized set based on LAVA support and/or convergent regulatory evidence. The strongest association mapped to the chr6p21.3/MHC region (rs389884), where four genes showed colocalization support, including *CLIC1* in whole blood and *C4A* in spleen. Because the chr6p21.3/MHC rs389884 region lead variant was unavailable for replication and no suitable proxy was identified, this signal was interpreted as an emerging candidate for functional validation rather than a replicated causal signal. Seven available variants replicated with concordant effects. An exploratory Roadmap immune chromatin-state overlap analysis placed 15 of 45 non-MHC lead variants (33.3%) directly, and 34 of 45 (75.6%) within ±10 kb, in active immune enhancer/promoter states. Pathway analyses highlighted type I interferon, JAK–STAT signaling, cytokine regulation, and antigen presentation, while bivariate LAVA analyses supported shared local genetic architecture with rheumatoid arthritis, systemic sclerosis, and Sjögren syndrome.

**Conclusions:**

This integrative post-GWAS analysis refines SLE association signals into biologically interpretable candidate regions and supports interferon and JAK–STAT signaling as central genetically supported pathways in SLE.

## Highlights

• Meta-analysis identified 46 genome-wide significant SLE-associated loci.
• LAVA prioritized nine high-confidence candidate SLE regions.
• Bivariate LAVA analysis supported shared local genetic architecture with related autoimmune traits.
• Colocalization and immune chromatin-state overlap supported immune-tissue regulation at selected loci.
• Prioritized signals converged on interferon and JAK–STAT signaling pathways.

## Graphical abstract

**Graphical abstract. Integrative post-GWAS framework for prioritizing immune regulatory pathways and candidate effector signals in SLE**.(a) European-ancestry SLE GWAS summary statistics from Bentham et al. and FinnGen M13_SLE were combined using fixed-effects inverse-variance weighted meta-analysis. Independent locus identification identified 46 genome-wide significant loci.(b) Post-GWAS prioritization included independent replication in the Julià et al. Spanish cohort, Bayesian colocalization with GTEx v8 whole blood and spleen eQTLs, and functional pathway annotation. Seven available discovery-prioritized variants replicated with concordant effects, while the chr6p21.3/MHC region showed colocalization support for multiple genes, including *CLIC1* in whole blood and *C4A* in spleen.(c) Prioritized loci converged on type I interferon, JAK–STAT/cytokine signaling, and antigen-presentation pathways, highlighting shared autoimmune architecture and established immune-pathway therapeutic candidates.

**Figure:**
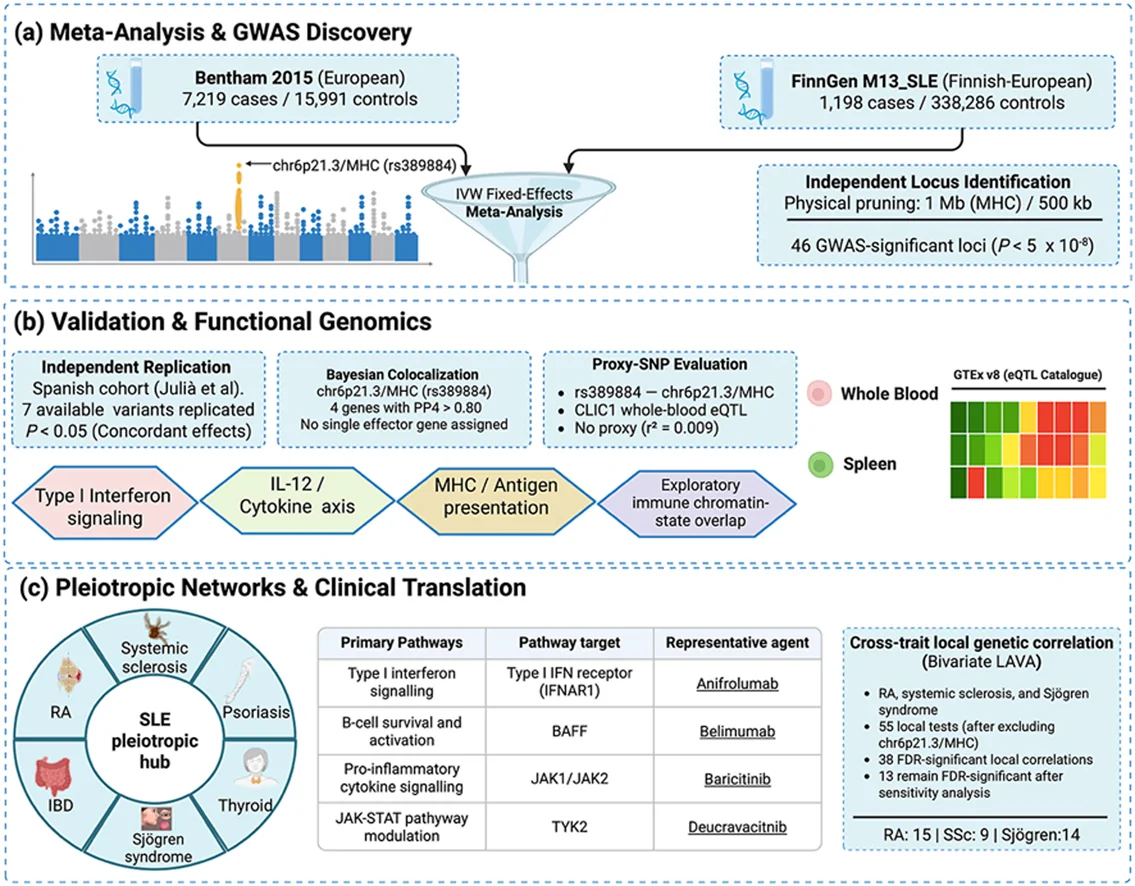

## 1 Introduction

Systemic lupus erythematosus (SLE) is a complex, heterogeneous polygenic autoimmune disease characterized by dysregulated innate and adaptive immune responses, leading to the production of autoantibodies, chronic inflammation, and multi-organ damage [[1], [2], [3]]. In SLE, genetic, environmental, and hormonal factors disrupt innate and adaptive immunity, causing loss of B- and T-cell tolerance, autoantibody production, and inflammatory cytokine responses that drive tissue damage [4,5]. Despite significant advancements in the development of targeted therapies, including those directed at type I interferon and B-cell signaling pathways, a substantial proportion of patients fail to achieve sustained remission, and mortality remains elevated compared to the general population [6,7]. These limitations underscore the need for a deeper, mechanism-driven understanding of disease biology to enable precision therapeutic strategies.

Over the past decade, genome-wide association studies (GWAS) have successfully mapped the genetic architecture of SLE, implicating hundreds of susceptibility loci [8], highlighting key biological nodes, such as interferon signaling, innate immune sensing, and adaptive immune activation [9,10]. However, the field has reached a translational plateau: while statistical associations are abundant, they rarely translate into mechanistic insights [11]. Most GWAS signals reside in non-coding regions and are embedded within complex linkage disequilibrium (LD) structures, particularly in immune-dense regions such as the major histocompatibility complex (MHC) [12,13]. As a result, distinguishing causal variants, identifying effector genes, and linking association signals to biological function remain major challenges [2].

This gap between statistical association and biological interpretation represents a critical bottleneck in post-GWAS research. While many loci are reproducibly associated with SLE risk, comparatively few can be confidently prioritized as mechanistically relevant or suitable for therapeutic follow-up. Addressing this challenge requires integrative approaches that combine genetic association data with functional genomics, regulatory annotation, and tissue-specific gene expression information.

In parallel, SLE exhibits a genetic architecture that overlaps significantly with other autoimmune and inflammatory disorders such as rheumatoid arthritis, Sjögren syndrome, and systemic sclerosis, highlighting common pathways of immune dysregulation and pleiotropy [14,15]. This alignment supports the clinical relevance of targeted therapies affecting interferon and JAK–STAT pathways [[16], [17], [18]]. Despite these advances, it remains unclear which SLE-associated loci are the most reproducible, biologically interpretable, and clinically relevant after systematic downstream prioritization.

To address this gap, we applied an integrative post-GWAS framework to refine the genetic architecture of SLE in European populations. By combining large-scale meta-analysis with local genetic architecture analysis (LAVA), Bayesian colocalization using tissue-specific eQTL data, independent replication, pathway enrichment, and therapeutic target prioritization, we aimed to move beyond locus discovery toward identification of high-confidence candidate regions and regulatory signals. Rather than treating all genome-wide significant loci as equally informative, this approach prioritizes variants and regions supported by convergent statistical, regulatory, and functional evidence. Through this framework, we refine a broad set of associated loci into a focused subset of high-confidence candidates, providing a more mechanistically informed view of SLE susceptibility. These findings contribute to a deeper understanding of immune dysregulation in SLE and offer a foundation for future functional validation and targeted therapeutic development.

## 2 Methods and materials

### 2.1 Study design and analytical overview

We applied an integrative post-GWAS pipeline to identify, refine, and functionally characterize loci associated with SLE susceptibility. The study was designed as a two-stage analysis consisting of a discovery meta-analysis followed by independent validation. The discovery stage integrated two European-ancestry GWAS summary-statistic datasets, FinnGen and Bentham et al., whereas the validation stage used the Spanish SLE cohort from Julià et al. only. The workflow consisted of six sequential stages: (i) inverse-variance weighted (IVW) fixed-effects meta-analysis of two extensive European SLE datasets; (ii) local signal refinement using Local Analysis of coVariant Associations (LAVA); (iii) Bayesian colocalization with tissue-specific eQTL data; (iv) independent replication in a Spanish SLE cohort; (v) functional interpretation through pathway enrichment and protein-protein interaction (PPI) networks; and (vi) therapeutic target prioritization. This study was not prospectively pre-registered. All analyses described are exploratory unless stated otherwise, and findings should be considered hypothesis-generating pending independent replication in additional European and non-European cohorts. The principal aim was to prioritize high-confidence, biologically plausible loci with translational relevance (Graphical abstract). An overview of the study workflow is provided in Supplementary Fig. 1.

### 2.2 Discovery GWAS datasets and quality control

Discovery analysis integrated summary statistics from two SLE GWAS datasets of European ancestry: FinnGen and Bentham et al. The FinnGen dataset was obtained from the FinnGen R12 M13_SLE and included 1198 SLE cases and 338,286 controls of Finnish ancestry. The Bentham et al. dataset included 7219 SLE cases and 15,991 controls of European ancestry. Together, these datasets formed the discovery meta-analysis, comprising 8417 SLE cases and 354,277 controls.

Per-study quality control was applied before meta-analysis. Variants were excluded if they had imputation quality INFO < 0.8, minor allele frequency MAF < 0.01, missing association statistics, missing or inconsistent allele information, or ambiguous strand orientation that could not be resolved using allele-frequency information. All variants were harmonized to a common genome build and aligned to the same effect allele before a fixed-effects inverse-variance-weighted meta-analysis.

The raw discovery meta-analysis included 7,419,322 variants. Filtering of the meta-analysis output excluded variants with missing required fields and variants lacking RSIDs, resulting in 6,782,131 SNPs retained for downstream reporting and summary-statistic release. The datasets used for discovery meta-analysis and independent replication are summarized in Table 1.

**Table 1:** **Summary of GWAS datasets used for discovery meta-analysis and independent replication in SLE.** The discovery stage integrated FinnGen and Bentham et al. summary statistics. The Spanish SLE cohort from Julià et al. was reserved for independent validation and was not included in the discovery meta-analysis.

Analysis stage	Dataset/Cohort	Population	Cases	Controls	Analytical purpose
Discovery meta-analysis	FinnGen R12 M13_SLE	Finnish (European)	1198	338,286	Primary discovery dataset
	Bentham et al. (2015)	European	7219	15,991	Primary discovery dataset
Combined meta-analysis	FinnGen + Bentham et al.	European	8417	354,277	Fixed-effects IVW meta-analysis
Independent replication	Julià et al. (2018)	Spanish/Iberian	907	1524	Independent validation of variants

### 2.3 Independent replication cohort

Independent validation was performed using the Spanish SLE GWAS cohort from Julià et al. This cohort comprised 907 SLE cases and 1524 Spanish controls. The Julià Spanish cohort was not included in the discovery meta-analysis and was used only for validation.

Discovery-prioritized lead variants were tested for validation in the Spanish cohort, where available. Validation was defined as nominal significance (P < 0.05) together with a concordant direction of effect between the discovery meta-analysis and the Spanish validation cohort. Variants unavailable in the validation dataset were reported as unavailable rather than non-validated. Where suitable proxy variants were available, proxies were selected using European linkage disequilibrium structure with r^2^ ≥ 0.8.

### 2.4 Harmonization and meta-analysis

GWAS summary statistics from FinnGen and Bentham et al. were harmonized before meta-analysis. Variant identifiers, chromosome positions, effect alleles, non-effect alleles, effect estimates, standard errors, P-values, and allele frequencies were standardized across datasets where available. Ambiguous A/T and C/G variants were excluded unless allele frequencies enabled reliable strand alignment.

Meta-analysis was performed using an inverse-variance weighted fixed-effects model, with effect estimates aligned to the same effect allele across studies. The Bentham et al. cohort exhibited a per-study genomic inflation factor of λGC = 1.409; genomic control correction was applied to these summary statistics prior to meta-analysis. Heterogeneity between discovery datasets was assessed using Cochran's Q statistic and I^2^. Genome-wide significance was defined as P < 5 × 10^−8^.

Genome-wide significant variants were grouped into independent loci using European linkage disequilibrium reference data. Non-MHC loci were defined using a 500-kb window around the lead variant. Because of the extended and complex linkage disequilibrium structure of the MHC region, chr6:25–34 Mb was treated separately and interpreted with additional caution.

### 2.5 Local genetic architecture analysis

Regional refinement of associated loci was performed using LAVA v0.1.0 [19]. Each genome-wide significant lead variant was mapped to its corresponding linkage disequilibrium (LD) block using the 1000 Genomes Phase 3 European reference panel. LAVA evaluated local genetic architecture and prioritized loci with robust regional associations. Since LAVA assesses regional architecture rather than causal variants, prioritized loci were interpreted as high-confidence candidate regions rather than definitive causal signals. Loci meeting local association criteria were carried forward for colocalization, validation, and functional analyses.

Variant-level interpretation within LAVA-prioritized loci was based on discovery association strength, local genetic architecture, Bayesian colocalization evidence, and statistical fine-mapping. Ninety-five percent credible sets were derived with susieR [20] using the 1000 Genomes Phase 3 European reference panel. LAVA-prioritized regions were interpreted as candidate regions, and COLOC PP4 > 0.80 was used as evidence consistent with shared regulatory signal between SLE risk and immune-tissue gene expression. These analyses were interpreted cautiously, particularly in the MHC region where extended linkage disequilibrium limits causal resolution.

### 2.6 Bayesian colocalization with immune-eQTLs

Colocalization between SLE associations and regulatory signals was assessed at all 46 genome-wide significant loci where eQTL data were available, using COLOC v5.2 under a Bayesian framework (p1 = p2 = 1 × 10-4; p12 = 1 × 10-5). Complete nominal eQTL summary statistics were obtained from the eQTL Catalogue [21] for GTEx v8 whole blood (n = 670) and spleen (n = 227). Full regional summary statistics, including non-significant variants, were used as required by coloc.abf. For each locus, variants within the predefined regional window around the discovery lead variant were extracted and harmonized between the GWAS and eQTL datasets. Strong colocalization evidence was defined as PP4 > 0.80 and was interpreted as evidence consistent with a shared association signal between SLE risk and gene expression, not as definitive proof of causality. This caution was particularly important for the chr6p21.3/MHC region, where extended linkage disequilibrium can complicate interpretation of shared association signals [[22], [23], [24]].

### 2.7 Replication and effect-size concordance

Discovery-prioritized lead variants were tested in the independent Spanish validation cohort from Julià et al. Replication/validation required both nominal significance, P < 0.05, and concordant effect direction relative to the FinnGen–Bentham discovery meta-analysis.

Effect-size concordance between discovery and validation was evaluated using Pearson correlation of beta estimates. Forest plots were generated to visualize the consistency of effect estimates across FinnGen, Bentham et al., and the Spanish validation cohort. Variants unavailable in the validation cohort were not interpreted as failed validations; instead, they were reported separately as unavailable or proxy-tested where appropriate.

### 2.8 Sensitivity analyses

Robustness of meta-analysis results was evaluated by comparing fixed-effects and DerSimonian–Laird random-effects models across lead loci. Signals were considered stable if effect directions were concordant and random-effects estimates showed limited deviation or retained genome-wide significance.

Since the MHC region has extensive linkage disequilibrium and contains multiple immune-relevant genes, signals within chr6:25–34 Mb were interpreted cautiously. The chr6p21.3/MHC rs389884 region association was therefore treated as a candidate regulatory signal supported by statistical and regulatory evidence, rather than as definitive proof of the causal gene.

For loci not evaluated in the Spanish cohort, post hoc power and variant availability were assessed to distinguish limited statistical power or missing variant overlap from lack of replication.

### 2.9 Pathway enrichment analysis and protein-protein interaction (PPI) network analysis

Pathway enrichment was performed using fgsea and g:Profiler (FDR < 0.05) against MSigDB Hallmark, KEGG, Reactome, GO Biological Processes, and ImmuneSigDB gene sets. Statistical significance was assessed using false discovery rate correction, with FDR < 0.05 considered significant. Results were interpreted in the context of established lupus biology, including type I interferon signaling, JAK–STAT pathways, immune activation, and apoptosis.

Protein–protein interaction analysis was conducted using STRING v11.5 (interaction score > 0.7) [25], with network topology and hub genes identified using igraph [26] based on betweenness centrality. Network and pathway results were used for biological interpretation and therapeutic prioritization, rather than as independent evidence of causality.

### 2.10 Cross-trait local genetic correlation and regulatory chromatin-state overlap

Bivariate LAVA local genetic correlation was used to evaluate whether SLE-associated local genetic architecture was shared with three related autoimmune traits: rheumatoid arthritis, systemic sclerosis, and Sjögren syndrome. Analyses used the 1000 Genomes Phase 3 European reference panel and were restricted to LAVA blocks that could be modelled in both SLE and the comparator trait. The chr6p21.3/MHC block containing rs389884 was treated separately because complex LD in this region can produce unstable local-correlation estimates. Because the three comparator GWAS derive from FinnGen R12 and share control samples with the FinnGen component of the discovery meta-analysis, the bivariate analysis was repeated using Bentham et al., 2015 alone as the SLE input, a design in which sample overlap with the comparator cohorts is zero by construction; magnitudes from the primary analysis are therefore reported as upper bounds. To provide regulatory context, we also performed an exploratory Roadmap Epigenomics 15-state ChromHMM chromatin-state overlap analysis [27,28] of non-MHC loci using active immune enhancer/promoter annotations. This descriptive analysis was not treated as formal genome-wide histone-mark enrichment or partitioned heritability analysis. As an additional exploratory analysis, we also used LAVA's run.pcor function to test the conditional (partial) local genetic correlation between SLE and each secondary trait, conditioning on the remaining secondary trait(s) at the same locus (Supplementary Fig. 8; Supplementary Table 11).

### 2.11 Therapeutic target prioritization

High-confidence loci were mapped to candidate effector genes and integrated with pharmacological evidence from the Open Targets Platform and literature curation. Targets were ranked according to statistical support (meta-analysis P-value) and therapeutic maturity. Therapeutic target prioritization was interpreted as hypothesis-generating. Moreover, established immune pathways, including interferon and JAK–STAT signaling, were distinguished from emerging candidate targets requiring further functional validation. In particular, the chr6p21.3/MHC rs389884 region signal was treated as an emerging candidate regulatory signal, not as a validated therapeutic target for SLE.

Therapeutic mechanisms were annotated to highlight FDA-approved and investigational agents relevant to SLE or related autoimmune diseases.

### 2.12 Software and reproducibility

All analyses in this study were conducted using R v4.3.1 and Python v3.11. For high-performance data processing of genome-wide summary statistics, the data.table and vroom R packages were utilized. Downstream analytical tools included LAVA v0.1.0 for local genetic architecture and bivariate local genetic correlation analyses; coloc v5.2 for Bayesian colocalization; fgsea v1.26, g:Profiler, and clusterProfiler v4.8 for pathway enrichment; and igraph v1.5 together with STRINGdb v2.12 for protein–protein interaction network analysis.

To improve reproducibility and transparency, the full analytical workflow, input datasets, software, and key decision thresholds are summarized in Table 2. This table provides an overview of the sequential discovery, validation, functional annotation, and translational prioritization steps used in the study.

**Table 2:** Analytical workflow, software, and key parameters used in the integrative post-GWAS pipeline.

Analytical step	Input data	Main objective	Software/Resource	Key parameters
Discovery GWAS meta-analysis	FinnGen SLE GWAS and Bentham et al. European SLE GWAS	Identify SLE-associated variants in the discovery dataset	R v4.3.1; data.table; vroom; custom harmonization scripts	Fixed-effects inverse-variance weighted meta-analysis; genome-wide significance **P < 5 × 10^−8^**
Variant quality control and harmonization	GWAS summary statistics from FinnGen and Bentham et al.	Align alleles and retain high-quality variants	R v4.3.1; data.table; custom scripts	INFO ≥ 0.8; MAF ≥ 0.01; removal of ambiguous strand variants unless resolvable by allele frequency
Locus definition	Genome-wide significant discovery variants	Define independent SLE-associated regions	European LD reference panel; 1000 Genomes Phase 3	500-kb window for non-MHC loci; chr6:25–34 Mb treated separately as MHC region
Local genetic architecture analysis	Discovery meta-analysis loci	Prioritize regions with robust local association architecture	LAVA v0.1.0	LD blocks based on 1000 Genomes Phase 3 European reference panel
Bayesian colocalization	Discovery GWAS loci and eQTL Catalogue GTEx v8 nominal eQTLs	Identify loci sharing regulatory signals with gene expression	coloc v5.2; eQTL Catalogue GTEx v8 whole-blood and spleen nominal summary statistics	Immune-relevant tissues: whole blood and spleen; strong evidence defined as **PP4 > 0.80**
Variant-level regulatory prioritization	Nine LAVA-prioritized loci and 1000 Genomes European LD reference	Refine prioritized loci into 95% credible sets and prioritize candidate regulatory signals	susieR v0.12.35; COLOC v5.2; 1000 Genomes Phase 3 EUR LD reference	Statistical fine-mapping into 95% credible sets; COLOC PP4 > 0.80; LAVA-prioritized loci interpreted as candidate regions
Independent validation	Julià et al. Spanish SLE GWAS cohort	Test reproducibility of discovery-prioritized variants	R v4.3.1; custom scripts	Validation defined as concordant effect direction and **P < 0.05**; proxies allowed if **r^2^ ≥ 0.8**
Effect-size concordance	Discovery and validation beta estimates	Compare genetic effect estimates across datasets	R v4.3.1; ggplot2; cowplot	Pearson correlation of beta estimates; forest plots for lead variants
Pathway enrichment analysis	Candidate genes mapped from prioritized loci	Identify enriched immune and biological pathways	fgsea; g:Profiler; clusterProfiler	MSigDB Hallmark, KEGG, Reactome, GO Biological Process, ImmuneSigDB; **FDR < 0.05**
Protein-protein interaction network analysis	Candidate genes mapped from prioritized loci	Identify connected immune networks and hub genes	STRING v11.5; STRINGdb v2.12; igraphv1.5	STRING interaction score > 0.7; hub genes ranked by betweenness centrality
Cross-trait genetic correlation	SLE discovery meta-analysis and external trait GWAS summary statistics	Estimate shared genetic architecture with immune-mediated traits	Bivariate LAVA	1000 Genomes Phase 3 EUR LD reference; RA, systemic sclerosis, and Sjögren syndrome
Exploratory chromatin-state overlap	SLE GWAS summary statistics and functional annotations	Provide descriptive regulatory context using Roadmap immune-cell chromatin states	R v4.3.1; GenomicRanges; rtracklayer; Roadmap Epigenomics 15-state ChromHMM	Exploratory Roadmap immune-cell enhancer/promoter chromatin-state overlap; formal histone-mark enrichment reserved for future work
Therapeutic target prioritization	Candidate genes, colocalization results, pathway evidence, drug databases	Prioritize clinically relevant and emerging therapeutic targets	Open Targets Platform; literature curation	Integrated genetic support, regulatory evidence, pathway relevance, and therapeutic maturity
Visualization and reproducibility	Results from all analytical stages	Generate figures and ensure reproducibility	R v4.3.1; Python v3.11; ggplot2; cowplot; GitHub; Zenodo	Custom figure generation; code and summary statistic deposition.

### 2.13 Ethical considerations and data availability

This study used publicly available GWAS summary statistics from FinnGen (accessed via finngen.fi under open-access terms, release R12), Bentham et al. (GWAS Catalog accession GCST003156), and Julià et al. The original data collections were approved by the relevant institutional review boards, and participants provided informed consent as described in the respective primary publications. No additional ethics approval was required for this secondary summary-statistic analysis.

The GWAS meta-analysis summary statistics generated in this study are deposited on Zenodo (https://doi.org/10.5281/zenodo.20514335) [29]. All analysis scripts are maintained on GitHub public repository: https://github.com/vijayachitrabio/SLE_MetaAnalysis.

## 3 Results

### 3.1 Discovery meta-analysis quality control and study overview

After harmonization and quality control, summary statistics from FinnGen and Bentham et al. were combined in the discovery meta-analysis. The final discovery dataset included 8417 SLE cases and 354,277 controls. FinnGen contributed 1198 cases and 338,286 controls of Finnish ancestry, while Bentham et al. contributed 7219 cases and 15,991 controls of European ancestry.

The raw discovery meta-analysis included 7,419,322 variants. Filtering of the meta-analysis output excluded variants with missing required fields and those lacking RSIDs, resulting in 6,782,131 SNPs retained for downstream reporting and summary-statistic release.

Variants were retained for meta-analysis if they passed dataset-level quality control, had complete association statistics, showed consistent allele annotation, and could be aligned to the same effect allele across both discovery datasets. Variants with low imputation quality, low minor allele frequency, missing effect estimates, missing allele information, or unresolved strand ambiguity were excluded before meta-analysis.

**Fig. 1:**
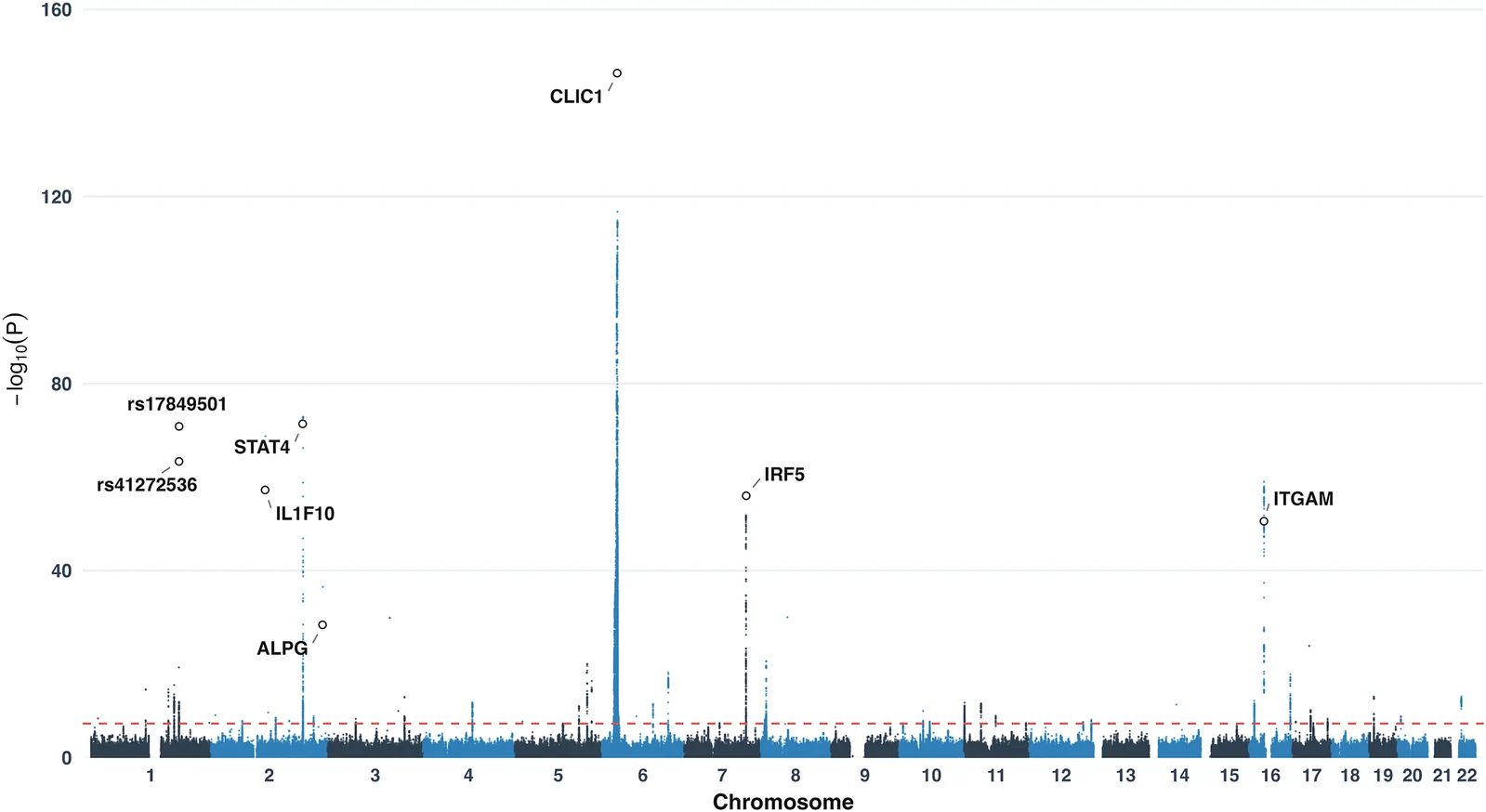
**Genome-wide meta-analysis of SLE identifies 46 genome-wide significant susceptibility loci.** Manhattan and Q-Q plots of the FinnGen–Bentham SLE discovery meta-analysis (8417 cases; 354,277 controls). The red horizontal line denotes genome-wide significance (P < 5 × 10^−8^). The strongest signal mapped to the chr6p21.3/MHC region near *CLIC1*. The QQ plot shows deviation in the tail, consistent with polygenic signal enrichment.

Fixed-effects inverse-variance weighted meta-analysis identified 46 genome-wide significant SLE-associated loci at P < 5 × 10^−8^. Genome-wide significant discovery loci are provided in Supplementary Table 1. The strongest association mapped to the chr6p21.3/MHC region near *CLIC1*, with additional significant signals observed at established SLE susceptibility loci, including *STAT4, IRF5, CLEC16A, STAT1, and TRIM21.* The genome-wide distribution of association signals and meta-analysis calibration are shown in Fig. 1.

**Fig. 2:**
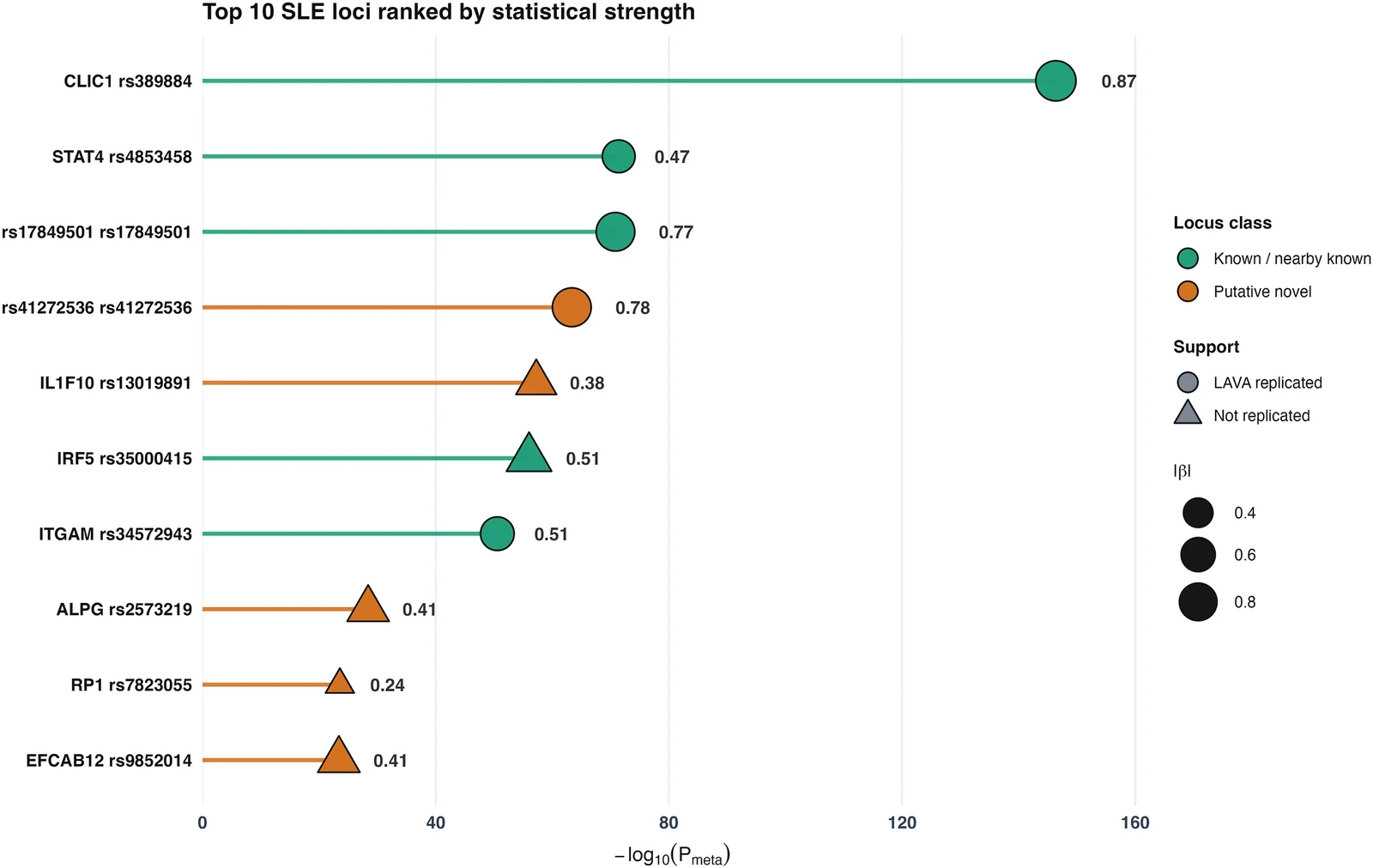
**Discovery association strength and LAVA support at the ten most strongly associated SLE loci.** Loci are ranked by discovery meta-analysis significance (−log_10_(Pmeta)). Marker colour denotes locus class (known or nearby-known versus putatively novel); marker shape denotes whether the locus met the LAVA candidate-region threshold (ρ > 0.30, PLAVA < 0.05); marker size and the adjacent value denote the absolute effect size |β|. Loci retained for downstream colocalization, replication assessment, functional annotation, and translational interpretation are listed in Table 3 and Supplementary Table 3.

Due to the extended linkage disequilibrium and dense immune-gene content of the MHC region, associations within chr6:25–34 Mb were retained but interpreted cautiously in all downstream analyses (see Fig. 2). In particular, the chr6p21.3/MHC rs389884 region signal was treated as a candidate regulatory signal rather than definitive evidence that *CLIC1* is the causal gene.

Meta-analysis calibration was assessed using genomic inflation and QQ-plot inspection. The QQ plot showed deviation primarily in the extreme tail, consistent with true polygenic enrichment and strong immune-associated loci rather than broad residual confounding. The combined meta-analysis genomic inflation factor was λGC = 0.917, indicating well-controlled residual inflation (Supplementary Fig. 2).

### 3.2 LAVA prioritization identifies candidate high-confidence loci

To refine the 46 genome-wide significant loci into regions with more interpretable local genetic architecture, we performed LAVA-based regional prioritization. Fourteen index variants met the predefined LAVA candidate-region threshold of ρ > 0.30 and PLAVA < 0.05, mapping to 12 unique LAVA-defined genomic blocks (Supplementary Table 3). Of these, nine primary Table 3 entries were carried forward for replication, pathway enrichment, and therapeutic interpretation: two with convergent Bayesian colocalization evidence in GTEx v8 whole-blood and/or spleen eQTLs (PP4 > 0.80) and seven LAVA-prioritized-only signals without colocalization support, labelled accordingly in Table 3. The complete LAVA prioritization results, including additional LAVA-supported signals not presented as primary Table 3 entries, are provided in Supplementary Table 3.

**Table 3:** LAVA-prioritized SLE susceptibility loci with colocalization evidence tiers.

RSID	Candidate gene/region	Chr: position GRCh38	Meta-analysis P-value	ρLAVA	PLAVA	COLOC PP4	Evidence tier
rs389884	chr6p21.3/MHC rs389884 region	6:31,973,120	4.22 × 10^−147^	0.623	2.88 × 10^−13^	0.932	High-confidence: LAVA + COLOC
rs4853458	STAT4	2:191,094,763	4.08 × 10^−72^	0.773	4.77 × 10^−13^	NA	LAVA-prioritized only
rs17849501	1q25 shared LAVA block	1:183,573,188	1.45 × 10^−71^	0.731	2.89 × 10^−12^	NA	LAVA-prioritized only
rs41272536	1q25 shared LAVA block	1:183,471,396	4.55 × 10^−64^	0.731	2.89 × 10^−12^	NA	LAVA-prioritized only
rs34572943	ITGAM	16:31,261,032	2.69 × 10^−51^	0.708	2.84 × 10^−10^	NA	LAVA-prioritized only
rs6671847	FCGR2A/STAT1-region	1:161,509,020	7.61 × 10^−13^	1.000	1.60 × 10^−5^	NA	LAVA-prioritized only
rs10912578	TNFSF4-region	1:173,282,717	1.75 × 10^−13^	0.679	4.39 × 10^−4^	NA	LAVA-prioritized only
rs34208825	XKR6-region	8:11,157,106	2.44 × 10^−11^	0.991	5.88 × 10^−6^	0.876	High-confidence: LAVA + COLOC
rs12928726	CLEC16A	16:11,097,715	3.08 × 10^−9^	1.000	2.77 × 10^−4^	NA	LAVA-prioritized only

Note: Table 3 includes nine LAVA high-confidence loci: two with convergent LAVA + colocalization evidence (High-confidence: LAVA + COLOC tier) and seven that are LAVA-prioritized only (no colocalization support; NA in the COLOC PP4 column). At rs389884 the PP4 shown is for CLIC1 in whole blood; three further genes colocalize at this locus (LINC00243 PP4 = 0.974, whole blood; C4A PP4 = 0.962 and CCHCR1 PP4 = 0.932, spleen), so no single effector gene is assigned. rs17849501 and rs41272536 map to the same 1q25 LAVA-defined regional block and are shown separately as retained index variants. The CLEC16A locus (rs12928726) shows LAVA ρ = 1.000, consistent with a homogeneous signal across the two discovery cohorts (I^2^ = 0%, Phet = 0.95; Supplementary Table 2).

The prioritized loci included established immune regions near *STAT4, ITGAM, FCGR2A/STAT1, TNFSF4, CLEC16A, and XKR6*, supporting the ability of the prioritization framework to recover biologically relevant immune loci. The strongest LAVA-confirmed signal was rs4853458 near *STAT4* (P = 4.08 × 10^−72^; LAVA ρ = 0.77), followed by *ITGAM* (P = 2.69 × 10^−51^; LAVA ρ = 0.72). The chr6p21.3/MHC region signal at rs389884 also met the prioritization threshold (P = 4.22 × 10^−147^; LAVA ρ = 0.62), supporting its inclusion as a high-confidence candidate region. However, because this signal lies within the broader chr6p21.3/MHC region, it was interpreted cautiously as a candidate regulatory region rather than definitive evidence that *CLIC1* is the causal effector gene. Variant-level association statistics for prioritized and replication-assessed loci, including effect sizes, are provided in Supplementary Tables 1 and 5.

Overall, LAVA prioritization identified 14 LAVA-supported index variants across 12 LAVA-defined genomic blocks from 46 genome-wide significant loci, of which nine primary entries were retained for Table 3: two with LAVA + colocalization support and seven LAVA-prioritized-only signals, providing a focused set of loci for functional annotation and translational interpretation. Representative regional association plots for prioritized loci are shown in Supplementary Fig. 7.

Statistical fine-mapping with susieR refined the nine LAVA-prioritized loci into 95% credible sets, generating 26 credible sets comprising 319 candidate variants (Supplementary Table 13). Fine-mapping, replication and therapeutic annotation were carried out on the prioritized locus set as defined before the final colocalization update, which differs from Table 3 at two positions: rs13332649 (*IRF8*) and rs2647928 (*IL12A*) were analysed but are not primary Table 3 entries, whereas credible sets were not generated for rs41272536 (1q25) and rs34208825 (*XKR6*). Several loci contained multiple statistically distinct signals. At the chr6p21.3/MHC locus, ten credible sets were identified and rs693906 reached the highest posterior inclusion probability (PIP > 0.999), whereas the GWAS lead variant rs389884 had PIP = 0.231, independently indicating that this region harbours multiple distinct signals. Given the complex linkage disequilibrium of the MHC, these results are interpreted as statistical prioritization of candidate variants rather than definitive evidence of causality.

### 3.3 Bayesian colocalization prioritizes immune regulatory mechanisms

To evaluate whether prioritized SLE association signals shared regulatory architecture with gene-expression variation, Bayesian colocalization was performed using GTEx eQTL data from immune-relevant tissues, including whole blood and spleen.

Colocalization was assessed at all 46 genome-wide significant loci. Across 3008 gene-tissue tests, 35 reached PP4 > 0.80 across 14 loci (Supplementary Table 4). Several of these loci did not meet the LAVA high-confidence threshold yet showed regulatory colocalization support, including *BLK* (PP4 = 0.957, spleen), *UBE2L3* (PP4 = 0.957, whole blood) and *TYK2* (PP4 = 0.806, whole blood). These findings support regulatory mechanisms underlying a subset of SLE susceptibility signals and suggest that several genetic associations may act through tissue-relevant effects on gene expression rather than through coding variation alone. Full Bayesian colocalization results are provided in Supplementary Table 4.

**Fig. 3:**
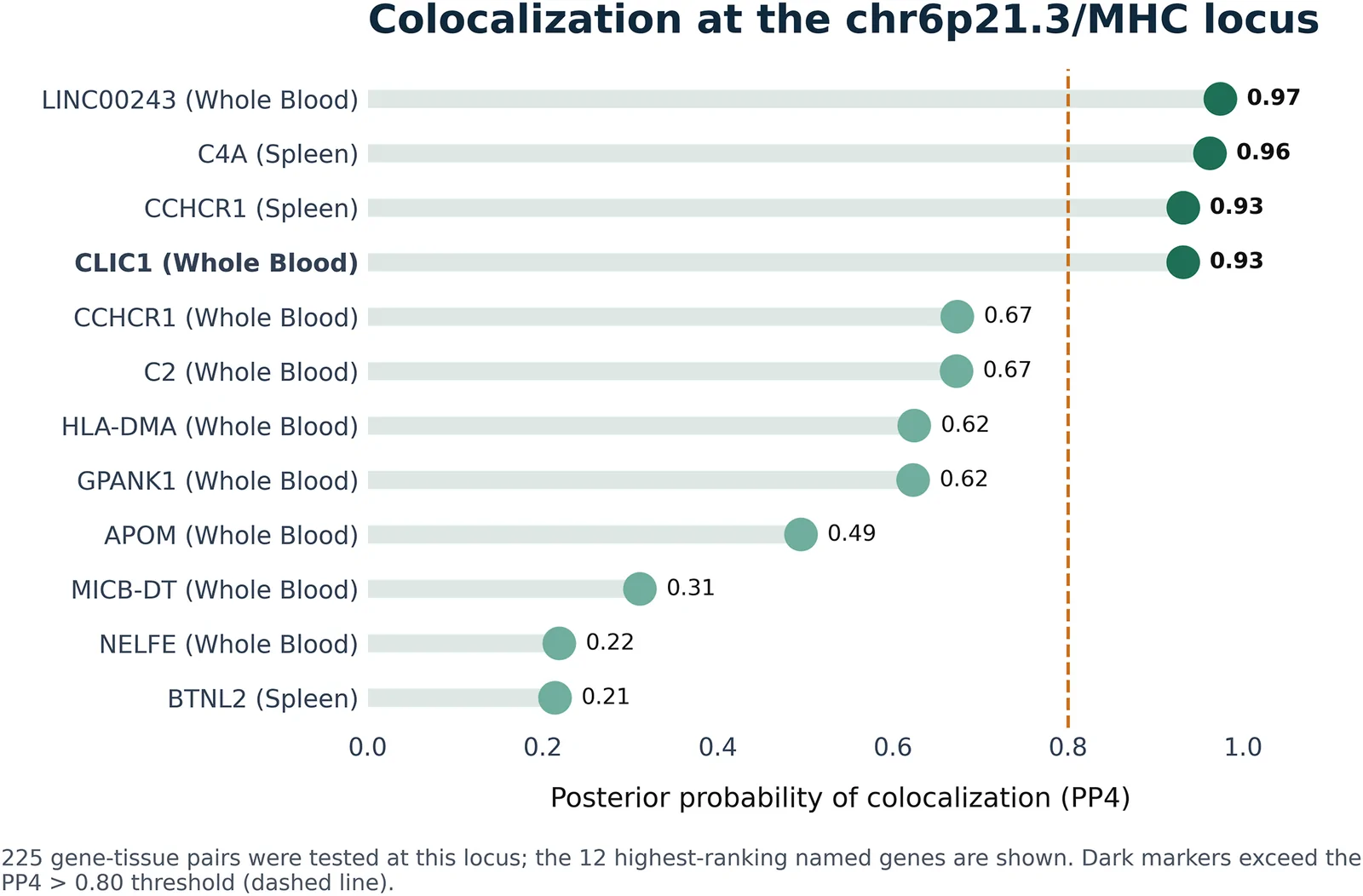
Colocalization at the chr6p21.3/MHC locus. Posterior probabilities of colocalization (PP4) between the SLE association signal at rs389884 and GTEx v8 eQTLs in whole blood and spleen, for the highest-ranking genes tested at this locus. Filled markers indicate PP4 > 0.80. Four genes exceeded this threshold — *LINC00243* (PP4 = 0.974) and *CLIC1* (PP4 = 0.932) in whole blood, and *C4A* (PP4 = 0.962) and *CCHCR1* (PP4 = 0.932) in spleen — consistent with the extended linkage disequilibrium of the MHC region, in which multiple genes can show comparable colocalization support. No single effector gene is assigned at this locus. Of 225 gene-tissue pairs tested here, the 12 highest-ranking named genes are shown; complete results are provided in Supplementary Table 4.

At the chr6p21.3/MHC locus (rs389884), four genes showed colocalization evidence: LINC00243 (PP4 = 0.974) and *CLIC1* (PP4 = 0.932) in whole blood, and *C4A* (PP4 = 0.962) and *CCHCR1* (PP4 = 0.932) in spleen (Fig. 3). *CLIC1* did not colocalize in spleen (PP4 = 0.159). This pattern is consistent with the extended linkage disequilibrium of the MHC region, in which multiple genes can show comparable colocalization support. Accordingly, no single effector gene can be assigned at this locus, and the region is interpreted as a candidate regulatory signal rather than definitive evidence for a causal effector gene. The colocalization of *C4A* is of particular interest given the established role of complement C4 in SLE. The second Table 3 locus with convergent colocalization support was rs34208825, where *NEIL2* colocalized in whole blood (PP4 = 0.876; Supplementary Table 4).

Colocalization support was not observed at *STAT4*, *ITGAM*, *IRF5*, *IRF8*, *CLEC16A* or *FCGR2A*, which are therefore retained as LAVA-prioritized loci on the basis of association strength and local genetic architecture rather than regulatory colocalization. These loci converge on immune pathways central to lupus pathogenesis, including interferon signaling and cytokine-mediated JAK–STAT activation.

### 3.4 Independent replication supports seven available prioritized loci

**Fig. 4:**
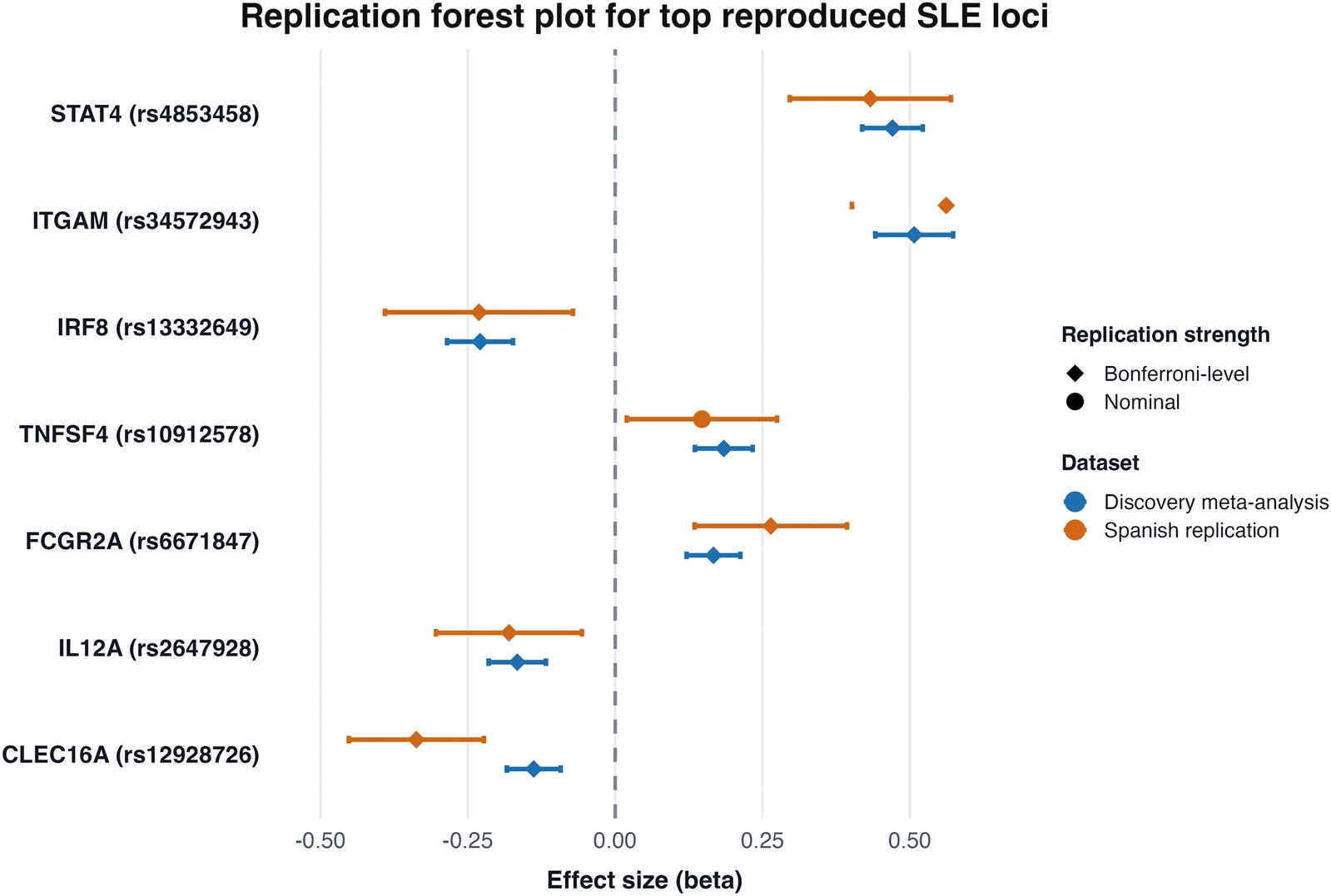
Effect-size concordance between discovery and replication cohorts. Comparison of effect sizes (β coefficients) between discovery (FinnGen–Bentham meta-analysis) and independent replication (Julià et al.) datasets for seven available discovery-prioritized variants. Strong concordance is observed (Pearson r = 0.969, 95% CI: 0.799–0.996, P = 0.000318, n = 7). The chr6p21.3/MHC rs389884 region lead variant was unavailable in the replication dataset and is not included.

Independent replication was performed in the Spanish SLE cohort from Julià et al., with 907 cases and 1524 controls. Of the discovery-prioritized regions, seven lead variants were available for replication testing. All seven variants showed concordant effect directions relative to the FinnGen–Bentham discovery meta-analysis and reached nominal significance in the Spanish cohort (replication P values ranged from P = 0.024 for rs10912578/*TNFSF4* to P = 5.84 × 10^−12^ for rs34572943/*ITGAM*; Supplementary Table 5). Discovery and replication effect estimates showed strong overall concordance (Pearson r = 0.969, 95% CI: 0.799–0.996, P = 0.000318, n = 7 variant pairs; Fig. 4). The chr6p21.3/MHC region lead variant rs389884 was unavailable for direct testing in the Spanish replication cohort and was not counted as replicated. A candidate proxy, rs3130346, showed very weak LD with rs389884 in the 1000 Genomes Phase 3 European reference panel (r^2^ = 0.009), far below the predefined r^2^ ≥ 0.8 proxy threshold, and was therefore not used as a proxy. The 1q25 index variants (rs17849501, rs41272536) were likewise not present in the Julià dataset. Replication was assessed for independent discovery leads defined by 500-kb physical pruning, a criterion that is not identical to the LAVA block definition used for Table 3: rs34208825 lies 336 kb from rs4840568 (*BLK*; discovery P = 1.09 × 10^−23^) and therefore fell inside that variant's window, so this region was tested under rs4840568, which replicated (P = 1.15 × 10^−4^, concordant direction). Supplementary Table 5 reports the status of every Table 3 entry. Per-variant β estimates, replication P values, and cohort-level heterogeneity statistics are provided in Supplementary Tables 2 and 5.

### 3.5 Sensitivity analysis results

Sensitivity analyses were performed to evaluate the robustness of the discovery meta-analysis and the influence of between-cohort heterogeneity. Fixed-effects and random-effects models produced broadly concordant results across the lead loci, with consistent effect directions for the major prioritized signals.

Evidence of heterogeneity was observed at a limited number of loci, including regions near *STAT4* (I^2^ = 95.2%), *ITGAM* (I^2^ = 91.9%), *IRF8* (I^2^ = 91.8%) and *TNFSF4* (I^2^ = 91.5%). However, these differences did not materially alter the interpretation of the principal prioritized immune loci, and the strongest associations remained supported in sensitivity analyses. Heterogeneity and cohort-level sensitivity statistics are provided in Supplementary Table 2 and Supplementary Fig. 3.

Signals within the MHC region were evaluated with caution because of extended linkage disequilibrium and dense immune-gene content. Although the chr6p21.3/MHC rs389884 region signal remained strongly supported in the discovery meta-analysis and showed immune-tissue colocalization evidence, it was not interpreted as definitive evidence of a single causal variant or effector gene.

For loci not evaluated in the Spanish replication cohort, variant availability was reviewed to distinguish missing overlap from lack of replication evidence (Supplementary Fig. 4). Unavailable variants were reported separately and not classified as failed replications.

### 3.6 Pathway enrichment and protein-interaction analyses reveal immune convergence

**Fig. 5:**
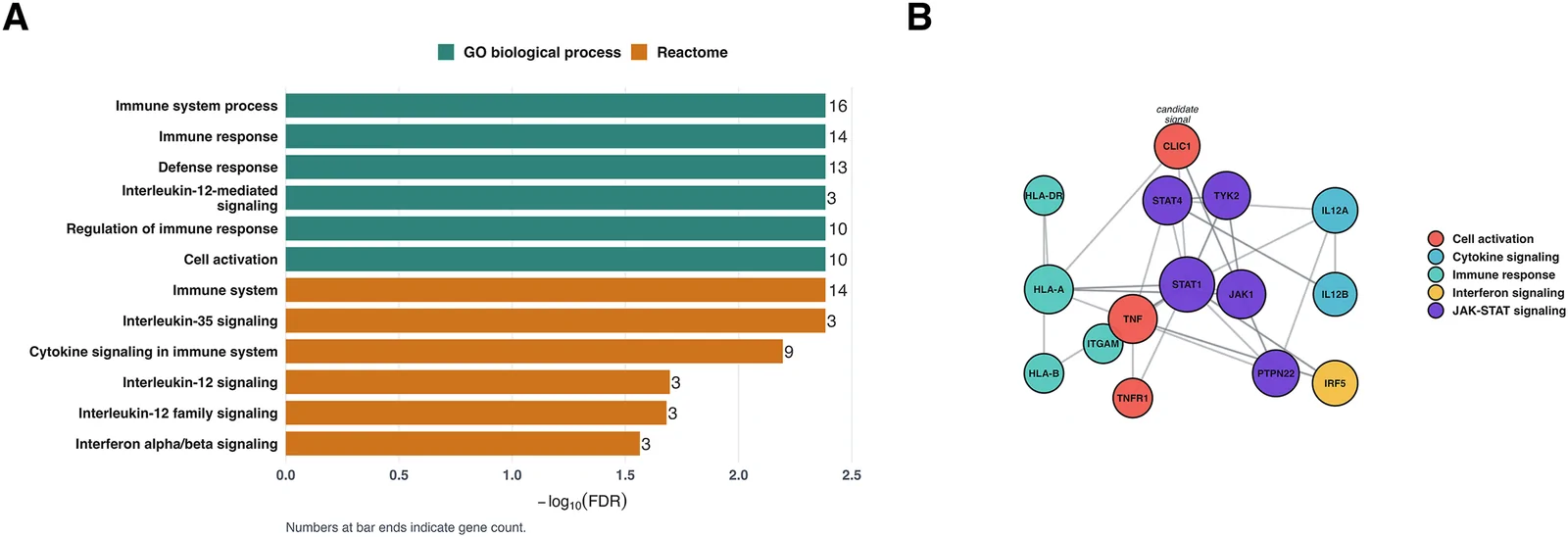
**Functional convergence of prioritized SLE-associated genes.** (A) Pathway enrichment analysis of genes mapped from prioritized SLE loci. Bars show enriched GO Biological Process and Reactome pathways ranked by −log_10_(FDR), with numbers indicating overlapping genes. Enriched terms mainly involved immune response, cytokine signaling, interleukin signaling, and interferon alpha/beta signaling. (B) Protein–protein interaction network of prioritized immune genes. Nodes represent candidate genes/proteins, edges indicate high-confidence functional interactions, and node colors denote major immune pathway categories. The network highlights convergence around *STAT1, STAT4, TYK2, JAK1, IRF5, IL12A, IL12B, HLA-A, HLA-B, HLA-DR, ITGAM, PTPN22, TNF, TNFR1, and CLIC1*.

Pathway enrichment analysis of prioritized SLE-associated genes showed convergence on immune pathways central to lupus pathogenesis. The strongest enrichment signals involved type I interferon signaling and JAK–STAT signaling (Fig. 5A), consistent with established mechanisms of innate immune activation and cytokine-mediated inflammation in SLE. Complete pathway enrichment results are provided in Supplementary Table 6.

Additional enriched pathways involved cytokine signaling, antigen presentation, immune-cell activation, and apoptosis-related processes. These results indicate that the prioritized loci do not represent isolated genetic signals, but instead converge on coordinated immune programs relevant to SLE susceptibility.

Protein–protein interaction analysis further supported this immune convergence. The interaction network contained densely connected modules centered on key immune regulators, including *STAT4, IRF5, JAK1, and JAK2* (Fig. 5B). These hubs connect genetic susceptibility signals to interferon-regulated transcriptional responses and cytokine signaling pathways with direct relevance to SLE pathogenesis.

Together, the pathway and network analyses support a model in which prioritized SLE loci converge on interferon-driven inflammation, JAK–STAT activation, antigen presentation, and adaptive immune-cell dysregulation.

### 3.7 Bivariate LAVA local genetic correlation supports shared autoimmune architecture

Bivariate LAVA local genetic correlation analysis demonstrated shared regional genetic architecture between SLE and related autoimmune diseases. Across 55 SLE cross-trait local tests after excluding the chr6p21.3/MHC block, 38 remained significant after FDR correction (FDR < 0.05), including 15 with rheumatoid arthritis, 9 with systemic sclerosis, and 14 with Sjögren syndrome (Supplementary Fig. 5; Supplementary Table 9). Several prioritized immune loci showed strong local sharing, including *STAT4* with rheumatoid arthritis (ρ = 0.734, FDR = 5.28 × 10^−14^), systemic sclerosis (ρ = 0.727, FDR = 5.56 × 10^−6^), and Sjögren syndrome (ρ = 0.584, FDR = 1.29 × 10^−9^). These results were interpreted as evidence of shared regional architecture rather than directional causal effects. Because the three comparator GWAS derive from FinnGen R12 and share control samples with the FinnGen component of the discovery meta-analysis, we repeated the analysis using Bentham et al., 2015 alone as the SLE input, in which sample overlap with the comparator cohorts is zero by construction (Supplementary Table 12). Local correlations were attenuated (mean ρ 0.501 to 0.244; 51 of 55 estimates decreased), and 13 locus-trait pairs remained significant after FDR correction in both analyses, including *STAT4* with all three traits. The direction of effect was preserved in 50 of 55 tests. Magnitudes from the primary analysis should therefore be interpreted as upper bounds, while the qualitative finding of shared local architecture is robust to sample overlap.

The chr6p21.3/MHC block containing rs389884 was excluded from cross-trait claims after sensitivity review, because complex MHC LD produced unstable local-correlation estimates. Therefore, *CLIC1* is presented as a discovery and immune-eQTL colocalization candidate, not as a cross-trait validated signal. As exploratory regulatory context, 15 of 45 non-MHC lead SNPs (33.3%) directly overlapped active immune enhancer/promoter chromatin states, and 34 of 45 loci (75.6%) overlapped these states within a±10 kb proximal regulatory-context window across Roadmap PBMC, B-cell, T-cell, and monocyte annotations (Supplementary Fig. 6; Supplementary Table 10).

As an additional exploratory analysis, we used LAVA's run.pcor function to test whether each pairwise SLE-secondary-trait local correlation remained when conditioning on the other secondary trait(s) at the same locus. Across 41 eligible non-MHC loci, 27 conditional (partial) correlation estimates were computable; all had 95% confidence intervals reaching the ±1 parameter boundary, reflecting limited power given the sample sizes of the secondary GWAS, and none survived FDR correction (minimum FDR = 0.072, *TNFSF4*/rs10912578 SLE-RA conditioned on systemic sclerosis, partial correlation = 0.71). The *STAT4* locus could not be evaluated because partial variances were negative for every target phenotype, consistent with a local signal shared broadly across all four traits rather than decomposable into a specific pairwise contribution. These conditional results are reported as an exploratory, appropriately caveated supplement (Supplementary Table 11) to the primary bivariate LAVA analysis above, which remains the adequately powered evidence for local cross-trait genetic sharing.

Together, the bivariate LAVA and exploratory chromatin-state overlap analyses indicate that prioritized SLE-associated loci are embedded within broader autoimmune local genetic architecture and frequently occur near immune regulatory annotations. Formal genome-wide histone-mark enrichment across disease-relevant immune-cell states remains an important follow-up analysis.

**Fig. 6:**
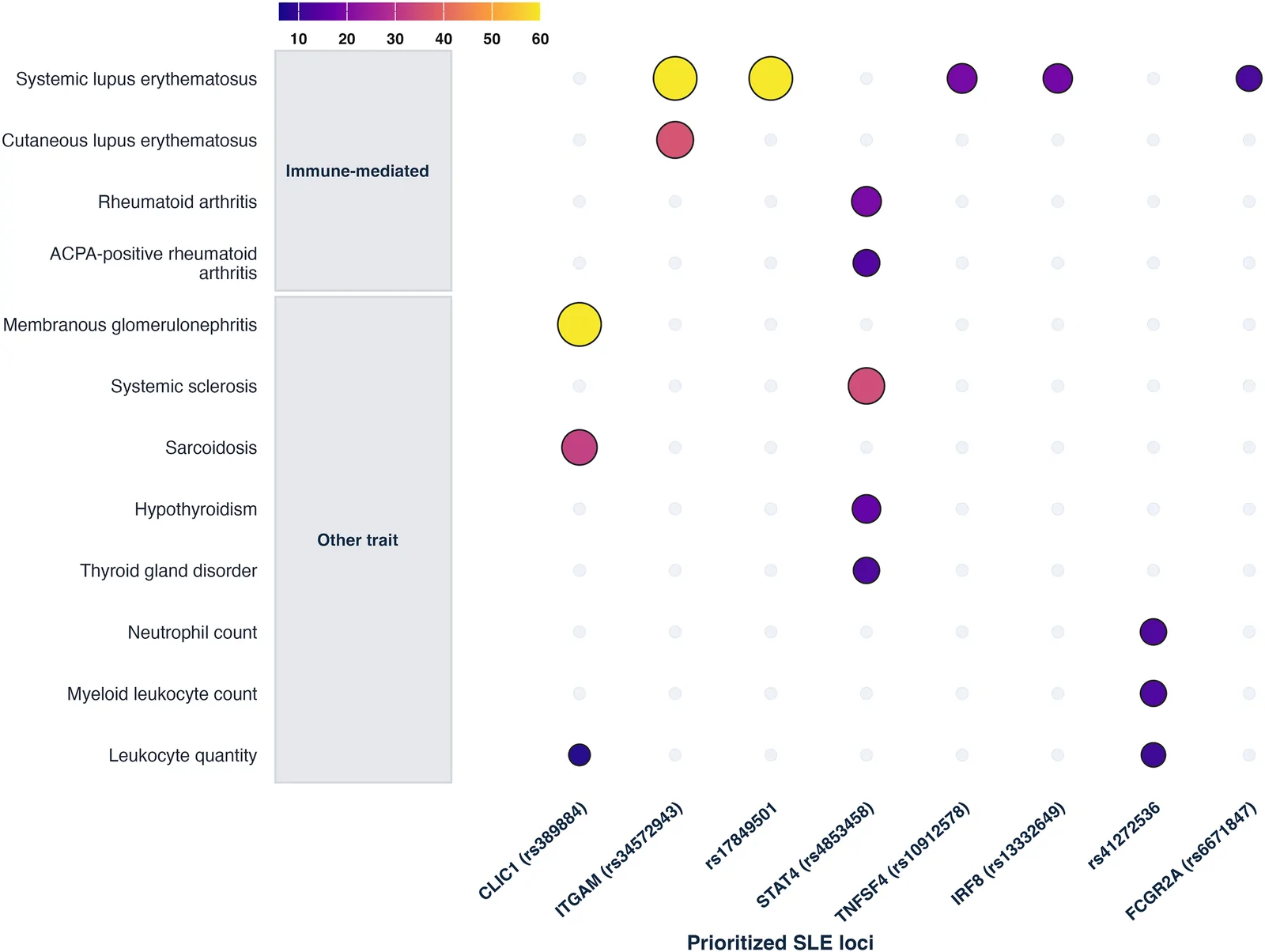
**Cross-trait associations between SLE loci and complex traits.** Bubble-matrix plot showing reported associations between prioritized SLE lead variants and other traits catalogued in the GWAS Catalog. Bubble size and colour indicate association strength (−log_10_ P); traits are grouped as immune-mediated or other. The strongest associations are observed at the chr6p21.3/MHC, *ITGAM*, and 1q25 loci. Full results are provided in Supplementary Table 7.

Cross-trait annotation of the prioritized lead variants against the GWAS Catalog identified 34 previously reported associations across nine variants, of which 13 were with immune-mediated traits and 21 with other traits (Fig. 6; Supplementary Table 7). The strongest immune-mediated associations were at the 1q25 (rs17849501, P = 3 × 10^−88^) and *ITGAM* (rs34572943, P = 9 × 10^−85^) loci, and the *STAT4* lead variant rs4853458 was additionally associated with rheumatoid arthritis (P = 3 × 10^−19^). The chr6p21.3/MHC lead variant carried the largest number of catalogued associations, including membranous glomerulonephritis (P = 8 × 10^−66^), consistent with the broad pleiotropy of this region. These catalogued associations summarise previously reported trait overlap and are distinct from the local genetic correlation analysis described above.

### 3.8 Therapeutic target prioritization identifies established and emerging immune candidates

**Fig. 7:**
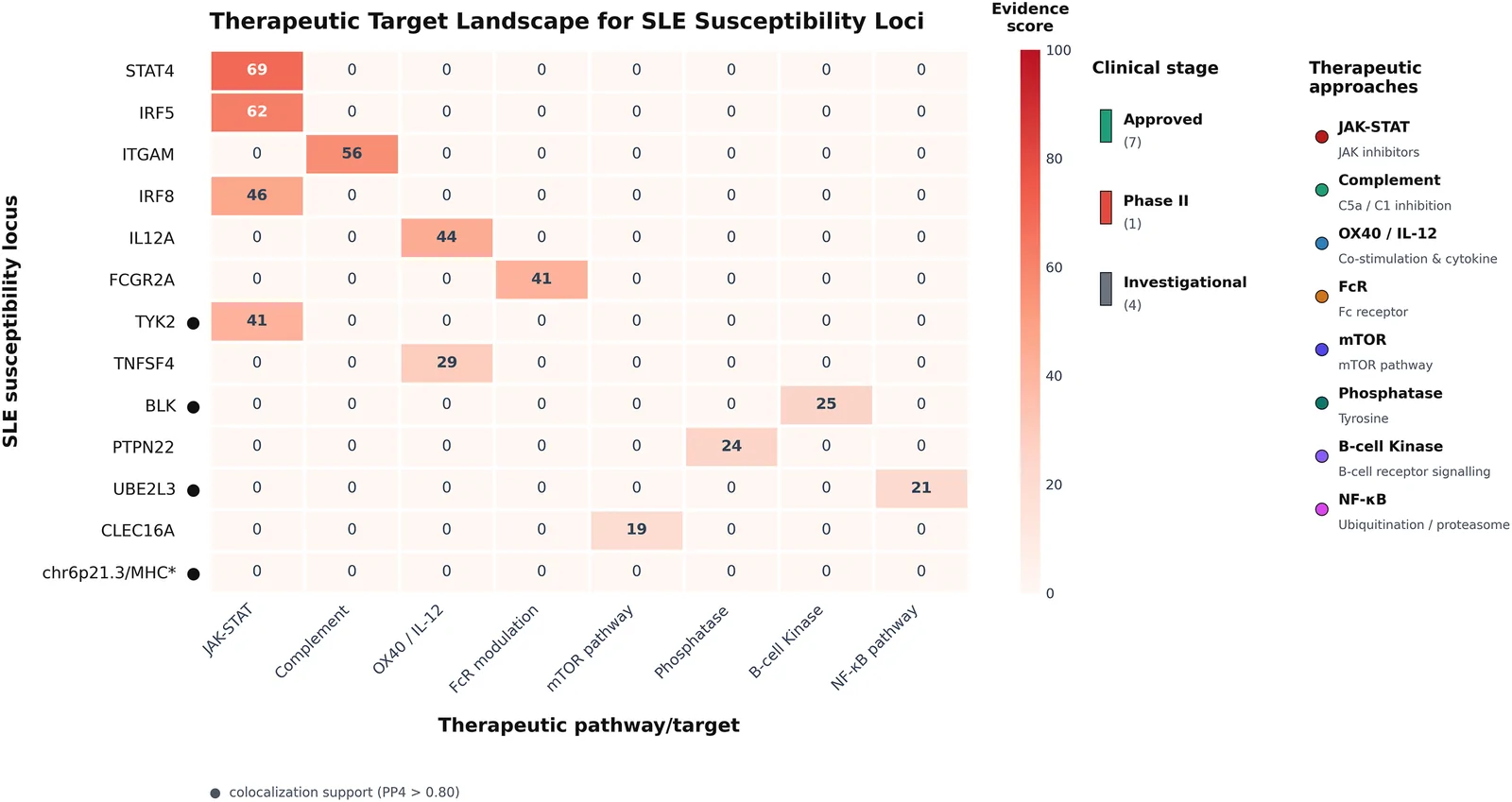
Therapeutic prioritization of candidate SLE targets. The heatmap illustrates how prioritized loci are ranked based on discovery association strength and stage in clinical development, mapped to their corresponding therapeutic pathway. The intensity of the color indicates the overall strength of the evidence, and filled circles beside locus labels denote colocalization support (PP4 > 0.80). Established pathway targets such as JAK–STAT cluster with strong support, whereas emerging candidates requiring functional validation are shown with lower evidence scores. Evidence score = 60 × min(1, −log_10_P/150) + 40 × therapeutic maturity (approved 1.0, phase III 0.8, phase II 0.6, investigational 0.4). chr6p21.3/MHC* scores 0 throughout because four genes colocalize at this locus with opposing effect directions, so no single therapeutic pathway is assigned (see Table 4).

Therapeutic target prioritization was performed by integrating genetic association strength, LAVA support, colocalization evidence, pathway membership, protein–protein interaction context, and pharmacological evidence. Prioritized targets were grouped by evidence tier (Table 4; Fig. 7). Therapeutic annotation details are provided in Supplementary Table 8.

**Table 4:** Prioritized therapeutic targets integrating genetic association, regulatory evidence, pathway context, and clinical development status.

Gene/target	Main support	Pathway	Clinical relevance	Evidence category	Notes
**JAK1/2**	Pathway/PPI convergence	IFN–JAK–STAT	JAK inhibitors evaluated in SLE and related immune diseases	Established pathway target	Pathway-level evidence
**TYK2**	Genetic/pathway support	IFN/cytokine signaling	TYK2 inhibitors relevant to immune-mediated disease	Established pathway target	SLE-specific efficacy requires context
**STAT4**	Discovery + LAVA support	STAT signaling	Established SLE susceptibility locus	Established immune candidate	Direct STAT4 therapy not established
**IRF5**	Genetic/pathway support	Type I IFN regulation	Established SLE susceptibility locus	Established immune candidate	Functional validation needed
**IL12A**	Genetic/pathway support	IL-12/Th1–Th17 signaling	IL-12/IL-23 pathway therapeutics exist in related diseases	Moderate pathway candidate	Indirect SLE relevance
**TNFSF4**	Genetic/pathway support	T-cell costimulation	OX40/OX40L axis under immune-therapy investigation	Moderate pathway candidate	Target-specific validation needed
**BLK**	Genetic/B-cell support	B-cell signaling	B-cell pathway relevance	Moderate immune candidate	No direct BLK therapy claimed
**ITGAM**	Genetic/immune support	Myeloid adhesion/complement-related response	Indirect relevance to inflammatory/complement pathways	Moderate immune candidate	Pathway-level mapping only
**FCGR2A/Fc pathway**	Immune/PheWAS support	Fc receptor signaling	Indirect relevance to Fc/FcRn biology	Supportive immune candidate	Not direct FCGR2A targeting
**chr6p21.3/MHC multi-gene region**	Discovery + LAVA + colocalization	MHC region; no single effector pathway assigned	Preclinical; functional validation required	Emerging candidate	Not replicated; no single effector gene assigned

The highest-priority established pathway targets included JAK1/2, TYK2, STAT4, and IRF5, reflecting convergence of genetic support with interferon and JAK–STAT pathway enrichment. These targets showed support across multiple analytical layers, including pathway enrichment and protein-interaction network analysis.

The chr6p21.3/MHC rs389884 region signal was also prioritized because it showed strong discovery association, LAVA support, and immune-tissue colocalization evidence. However, it was classified as an emerging candidate regulatory signal, rather than a validated therapeutic target, because it lies within the chr6p21.3/MHC region and was not available for direct replication in the Spanish cohort (Table 4; Fig. 7).

Overall, the prioritization analysis identified established immune-pathway targets and emerging candidate regulatory signals for downstream functional follow-up.

## 4 Discussion

This study applied an integrative post-GWAS framework to refine the genetic architecture of European-ancestry SLE. By combining meta-analysis, LAVA-based local genetic refinement, Bayesian colocalization with immune-relevant eQTL datasets, replication analysis, pathway enrichment, bivariate LAVA cross-trait analysis, and therapeutic prioritization, we identified 46 genome-wide significant loci, refined these to 14 LAVA-supported index variants across 12 LAVA-defined genomic blocks (Supplementary Table 3), and retained nine primary Table 3 entries, including two with LAVA + colocalization support and seven LAVA-prioritized-only signals. These prioritized loci collectively highlighted immune pathways centered on type I interferon signaling, JAK-STAT activation, cytokine regulation, and antigen presentation, reinforcing the central role of immune dysregulation in SLE pathogenesis.

A key strength of this study is its emphasis on biological prioritization rather than locus discovery alone. Although SLE is well established as a highly polygenic disease, the major challenge is identifying which loci are most reproducible, mechanistically interpretable, and translationally relevant [3,30]. By integrating multiple complementary analytical layers, our framework reduced the complexity of the genetic landscape and identified a focused set of high-confidence loci, thereby providing a more informative basis for downstream functional investigation [31].

Among the prioritized loci, the chr6p21.3/MHC region signal emerged as a prominent candidate, supported by strong association evidence at rs389884 (P = 4.22 × 10^−147^), LAVA-based local genetic support, and colocalization with immune-relevant regulatory signals in whole blood (*CLIC1* PP4 = 0.932), alongside colocalization of additional genes at the same locus (*LINC00243*, *C4A* and *CCHCR1*). Interpretation of this signal is particularly challenging because it resides within the extended MHC region, where dense linkage disequilibrium complicates causal inference [12,13]. Nevertheless, the consistency of evidence across association strength, local genetic architecture, and regulatory colocalization supports the chr6p21.3/MHC rs389884 region as a high-priority candidate for functional validation, while emphasizing the need for cautious interpretation [32].

Statistical fine-mapping added a variant-level layer to the LAVA-defined candidate regions, generating 26 credible sets across the nine prioritized loci. This was particularly informative at the chr6p21.3/MHC locus, where the credible-set structure and the colocalization of multiple neighbouring genes independently point to more than one underlying signal. Because fine-mapping depends on linkage disequilibrium structure, reference-panel accuracy, and summary-statistic quality, these results are interpreted as statistical prioritization of candidate variants rather than definitive causal inference.

Functionally, *CLIC1* encodes a Chloride Intracellular Channel 1 involved in oxidative stress responses, macrophage activation, inflammasome-associated inflammation, and intracellular ion homeostasis. Although not historically central to lupus genetics, these functions align closely with emerging models of lupus pathogenesis, emphasizing innate immune activation and inflammatory amplification. Colocalization at this locus was observed in whole blood for *CLIC1*, and in spleen for *C4A* and *CCHCR1*, consistent with regulatory effects in immune-relevant tissues. However, the present study does not establish *CLIC1* as the causal gene. Instead, the combined statistical, fine-mapping, and regulatory evidence support the chr6p21.3/MHC rs389884 region as a candidate locus requiring functional validation [1,11,16,[33], [34], [35]].

Beyond the chr6p21.3/MHC rs389884 region, prioritized loci and regions, including *STAT4*, *ITGAM*, *FCGR2A*/*STAT1*, *TNFSF4*, *XKR6*, and *CLEC16A*, converged on pathways linked to interferon signaling, cytokine regulation, immune activation, and antigen presentation [10,36,37]. Functional enrichment and protein-interaction analyses further highlighted type I interferon and JAK-STAT signaling as central components of the prioritized network, with hub genes including *STAT4, IRF5, JAK1*, and *JAK2* [16,38]. These findings are highly consistent with current understanding of lupus biology and provide additional genetic support for pathways already under active therapeutic investigation [[16], [17], [18],^39^].

The replication analysis further strengthened confidence in the prioritized loci. Seven lead variants were evaluated in an independent Spanish cohort [40], and all showed nominal replication with concordant effect direction, suggesting that key prioritized signals are reproducible across related European populations despite underlying genetic heterogeneity [41,42]. Importantly, the chr6p21.3/MHC rs389884 region lead variant was unavailable for direct testing in the Spanish cohort and was therefore not counted as replicated. The evaluated candidate proxy rs3130346 was not suitable (r^2^ = 0.009 with rs389884 in 1000 Genomes Phase 3 EUR), so *CLIC1* prioritization is based on convergent discovery, LAVA, and colocalization evidence rather than independent replication.

The study has translational implications. Specifically, integration of genetic prioritization with regulatory evidence, pathway enrichment, protein-network context, and therapeutic annotation highlighted established immune pathways with current pharmacological relevance, including JAK–STAT and interferon signaling. Targets such as JAK1/2, TYK2, STAT4, and IRF5 were prioritized because they connect genetic susceptibility signals with immune pathways already implicated in SLE or related autoimmune diseases [39]. In contrast, the chr6p21.3/MHC rs389884 region signal represents an emerging candidate requiring functional validation. Although CLIC1-related biology has been linked to oxidative stress, ion-channel activity, and inflammatory regulation, no validated SLE-directed therapeutic strategy currently follows from this signal. Therefore, CLIC1 should be viewed as a promising candidate for mechanistic follow-up rather than an established therapeutic target.

## 5 Limitations and future perspectives

Several limitations should also be acknowledged. First, the study relied on summary statistics and therefore cannot definitively establish causal variants or mechanisms without experimental validation [43,44]. Second, colocalization and LAVA analyses provide statistical prioritization rather than direct functional proof and remain dependent on local LD structure, reference panel, and tissue availability [[45], [46], [47]]. Third, the eQTL framework analyses were primarily limited to the GTEx whole-blood and spleen datasets and may not fully capture disease-relevant immune-cell states [48,49]. Fourth, the Roadmap Epigenomics analysis was a descriptive chromatin-state overlap of lead variants and proximal windows, not a formal genome-wide histone-mark enrichment or partitioned heritability analysis. Finally, the European ancestry focus improves analytical consistency but limits immediate generalizability to non-European populations [50].

Future studies incorporating single-cell and cell-state–specific regulatory datasets (including stimulated and disease-context atlases) will help clarify the precise immune-cell contexts through which prioritized loci exert effects [51]. Experimental functional perturbation, for example, CRISPR-based genomic edits or gene perturbation in relevant primary immune cells or *in vivo* models, will be important to validate causality and to test the mechanistic role of prioritized genes such as *CLIC1* in SLE pathogenesis [52].

## 6 Conclusion

This study refines the European-ancestry SLE genetic landscape into a smaller set of high-confidence, biologically interpretable candidate regions. By integrating GWAS discovery, LAVA, colocalization, replication, pathway analysis, bivariate LAVA cross-trait analysis, and therapeutic annotation, the study provides a mechanistically grounded view of SLE susceptibility. The findings reinforce the importance of type I interferon signaling, JAK–STAT biology, cytokine regulation, and immune-cell regulatory mechanisms. The chr6p21.3/MHC rs389884 region signal emerges as a candidate for functional validation rather than a validated causal gene or therapeutic target. Overall, these results provide a prioritized resource for future functional and translational studies in SLE.

## CRediT authorship contribution statement

**Vijayachitra Modhukur:** Writing – review & editing, Writing – original draft, Visualization, Validation, Supervision, Software, Resources, Project administration, Methodology, Investigation, Formal analysis, Data curation, Conceptualization. **Masuma Khatun:** Writing – review & editing, Writing – original draft, Visualization. **Naisarg Patel:** Writing – review & editing, Validation, Investigation. **Sajitha Lulu S:** Writing – review & editing. **Prakash Lingasamy:** Writing – review & editing, Investigation. **Andres Salumets:** Writing – review & editing, Supervision, Resources, Project administration, Funding acquisition.

## Ethics declaration

Written informed consent to take part in the original studies and to publish the article was obtained from all participants or their legal representatives as described in the original studies/data resources. The privacy rights of participants have been observed.

This study was performed in compliance with relevant laws, regulatory frameworks and guidelines where the research took place. This study was conducted in accordance with Declaration of Helsinki; local ethical and data-access approvals of the original GWAS studies/data resources. Ethics committee approval was not required under relevant laws and institutional guidelines. This study used only publicly available, de-identified GWAS summary statistics from FinnGen, Bentham et al., and Julià et al. No new participants were recruited, no individual-level data were accessed, and no new biological samples were collected. The original studies/data resources obtained ethics approvals and participant consent according to their respective institutional and national requirements. Therefore, no additional ethics committee approval was required for this secondary summary-statistic analysis.

## Declaration of generative AI use

During manuscript preparation, the authors used ChatGPT (GPT-5.5 Thinking, OpenAI) and Claude AI (Sonnet 4.6, Anthropic) for language refinement and code review. The authors reviewed and verified all AI-assisted content and take full responsibility for the final manuscript.

## Funding

This work was supported by 10.13039/501100002301Estonian Research Council grant (MOB3JD1246), 10.13039/501100003125Finnish Cultural Foundation, K. Albin Johanssons stiftelse.

## Declaration of competing interest

The authors declare that they have no known competing financial interests or personal relationships that could have appeared to influence the work reported in this paper.

## Data Availability

The GWAS meta-analysis summary statistics generated in this study are available through Zenodo at https://doi.org/10.5281/zenodo.20514335. Analysis scripts and reproducible workflow materials are available on GitHub at https://github.com/vijayachitrabio/SLE_MetaAnalysis. External datasets used in this study were obtained from FinnGen, Bentham et al. (GCST003156), and Julià et al. through their respective public resources and primary publications.
