# Supplementary material for: Integrative post-GWAS analysis prioritizes immune regulatory pathways and candidate effector signals in systemic lupus erythematosus
Source: J Transl Autoimmun. 2026 Sep 3;13:100399. doi: 10.1016/j.jtauto.2026.100399 (PMC13572754; doi:10.1016/j.jtauto.2026.100399)
Supplement: Supplementary file 1 [file mmc1.docx]

**Integrative Post-GWAS Analysis Prioritizes Immune Regulatory Pathways and Candidate Effector Signals in Systemic Lupus Erythematosus**

Vijayachitra Modhukur*^1,2^, Masuma Khatun^2,3^, Naisarg Patel^1^, Sajitha Lulu S^4^, Prakash Lingasamy^5,6^, Andres Salumets ^1,2,7^*

1. Celvia CC AS, Tartu, Estonia
2. Department of Obstetrics and Gynecology, Institute of Clinical Medicine, University of Tartu, Tartu, Estonia
3. Department of Obstetrics and Gynecology, Helsinki University Hospital and University of Helsinki, Helsinki, Finland
4. Integrative Multiomics Lab, School of Bio Sciences and Technology, Vellore Institute of Technology, Vellore, India
5. Laboratory of Precision and Nanomedicine, Institute of Biomedicine and Translational Medicine, University of Tartu, Tartu, Estonia
6. Nalam Biosciences OÜ, Tartu, Estonia
7. Division of Obstetrics and Gynaecology, Department of Clinical Science, Intervention and Technology (CLINTEC), Karolinska Institutet, and Karolinska University Hospital, Stockholm, Sweden

*Corresponding author(s):

Dr. Vijayachitra Modhukur, Department of Obstetrics and Gynecology, Institute of Clinical Medicine, University of Tartu, L. Puusepa 8, 50406 Tartu, Estonia. E-mail: [modhukur@ut.ee](mailto:modhukur@ut.ee);

Prof. Andres Salumets, Division of Obstetrics and Gynaecology, Department of Clinical Science, Intervention and Technology (CLINTEC), Karolinska Institutet, and Karolinska University Hospital, Stockholm, Sweden. Email: [andres.salumets@ki.se](mailto:andres.salumets@ki.se)

**Supplementary Figure 1. Study workflow for SLE susceptibility locus discovery and prioritization.** Discovery analyses integrated Bentham 2015 and FinnGen M13_SLE summary statistics using inverse-variance weighted meta-analysis, followed by locus refinement through LD pruning and LAVA local-architecture assessment. Functional prioritization incorporated COLOC with GTEx v8 whole-blood and spleen eQTLs (eQTL Catalogue), pathway enrichment, and exploratory immune chromatin-state overlap. Prioritized signals were evaluated through Spanish replication, proxy-SNP evaluation, and sensitivity analyses. Cross-trait and translational interpretation included bivariate LAVA local genetic correlation and therapeutic annotation, yielding genome-wide significant SLE loci, prioritized candidate regions, shared autoimmune architecture, and immune regulatory pathways for downstream biological interpretation.

**Supplementary Figure 2.** **Quantile–quantile (QQ) plot of the discovery meta-analysis.** Observed −log_10_(*P*) values are plotted against expected values under the null hypothesis of no association. Marked deviation from the diagonal in the upper tail indicates enrichment of genuine association signals. The combined meta-analysis genomic inflation factor (λ_GC_ = 0.917) indicates no residual inflation. The Bentham et al. cohort was subject to genomic control correction prior to meta-analysis owing to its per-study λ_GC_ of 1.409, consistent with the highly polygenic architecture of SLE and the large sample size of the discovery dataset.

**Supplementary Figure 3. Sensitivity analysis of heterogeneity across discovery cohorts .** Comparison of effect size estimates from the primary fixed-effects IVW model and the DerSimonian–Laird random-effects model across the top discovery loci. Between-study heterogeneity was quantified using Cochran's Q statistic and the I² index. All effect-size shifts between the two models remained below 15%, and effect directions were consistent throughout, indicating that the reported associations were robust to between-study variance. Loci showing I² > 50% (including TNFSF4 and IRF8) retained significance under the random-effects model.

**Supplementary Figure 4. Additional replication assessment in the Spanish SLE cohort.** Replication power analysis and replication status for seven prioritized loci available in the Spanish SLE cohort (907 cases and 1,524 controls). Bars show statistical power (%) for each available variant. All seven variants showed concordant effect directions and reached nominal significance (P < 0.05) in replication. Discovery and replication effect sizes were strongly correlated (Pearson r = 0.969, P = 0.000318, 95% CI: 0.799-0.996, n = 7), supporting the robustness and cross-population portability of the prioritized signal set.

**Supplementary Figure 5. Bivariate LAVA local genetic correlation across related autoimmune traits. Bubble heatmap of local genetic correlations between SLE loci and rheumatoid arthritis, systemic sclerosis, and Sjögren syndrome. Bubble color indicates correlation direction and magnitude, and size indicates statistical significance after FDR correction. The chr6p21.3/MHC block containing rs389884 was excluded because complex LD produced unstable local-correlation estimates. Because the comparator GWAS derive from FinnGen R12 and share control samples with the FinnGen component of the discovery meta-analysis, a zero-overlap sensitivity analysis was performed using Bentham et al. 2015 alone (Supplementary Table 12). Local correlations were attenuated (mean r 0.501 to 0.244) while direction and the core set of shared loci were preserved; magnitudes shown here should therefore be interpreted as upper bounds.**

**Supplementary Figure 6. Exploratory overlap of SLE-associated loci with immune enhancer/promoter chromatin states.** Bars show the percentage of non-MHC SLE loci overlapping active promoter or enhancer states from Roadmap Epigenomics 15-state ChromHMM annotations in PBMCs, B cells, T cells, and monocytes. Dark bars indicate exact lead-SNP overlap, and light bars indicate overlap within a ±10 kb proximal regulatory-context window. This analysis was intended to provide descriptive regulatory context and was not interpreted as formal genome-wide histone-mark enrichment.

|  |  |
| --- | --- |

**Supplementary Figure 7. Regional association plots for representative prioritized SLE loci.** Regional association plots are shown for representative prioritized loci: (A) chr6p21.3/MHC rs389884 region, (B) STAT4, (C) IRF5, and (D) CLEC16A. These loci were selected to illustrate the strongest discovery signal (chr6p21.3/MHC rs389884 region; rs389884, P = 4.22 × 10⁻¹⁴⁷), at which four genes showed colocalization evidence (LINC00243 PP4 = 0.974 and CLIC1 PP4 = 0.932 in whole blood; C4A PP4 = 0.962 and CCHCR1 PP4 = 0.932 in spleen), consistent with extended MHC linkage disequilibrium, established SLE susceptibility loci with strong JAK–STAT relevance (STAT4), an established interferon-related locus supporting core lupus biology (IRF5; rs35000415 did not meet the LAVA high-confidence threshold and did not show colocalization support in the corrected analysis, and is shown here as an established immune locus), and an autoimmune-relevant prioritized locus adding mechanistic diversity beyond the interferon/JAK signalling axis (CLEC16A). SNP −log_10_(*P*) values are colour-coded by linkage disequilibrium (r²) with the lead SNP. All genomic coordinates in the regional plots are on GRCh37/hg19. Note: all other supplementary tables and the main manuscript use GRCh38 coordinates; positions in these regional plots are not directly comparable to table entries without liftover.

**Supplementary Figure 8**. **Exploratory LAVA conditional (partial) local genetic correlation between SLE and related autoimmune traits.** Forest plot of partial local genetic correlations (LAVA run.pcor) between SLE and each secondary autoimmune trait (rheumatoid arthritis, systemic sclerosis, Sjögren syndrome), conditioned on the remaining secondary trait(s) at the same locus. Diamonds indicate nominal p < 0.05; circles indicate p >= 0.05. Point fill indicates the direction/magnitude of the partial correlation. No estimate survived FDR correction (minimum FDR = 0.072, TNFSF4 locus). Error bars reaching the ±1 parameter boundary (grey shaded bands) are shown for completeness; boundary-adjacent intervals are common in local genetic correlation analyses and occur in both the bivariate and conditional results. the STAT4 locus could not be evaluated because partial variances were negative for every target phenotype. This analysis is reported as an exploratory, appropriately caveated supplement to the primary bivariate LAVA analysis (Supplementary Figure 5, Supplementary Table 9); see also Supplementary Table 11.
